# Effect of Tooth Preparation Design on Fracture Resistance and Marginal Adaptation of Zirconia-Reinforced Lithium Silicate and 3D-Printed Overlays

**DOI:** 10.3390/polym18030352

**Published:** 2026-01-28

**Authors:** Bülent Kadir Tartuk, Eyyüp Altıntaş, Mustafa Caner Akgül

**Affiliations:** 1 Department of Prosthodontics, Faculty of Dentistry, Bingöl University, Bingöl 12000, Türkiye; 2 Department of Prosthodontics, Faculty of Dentistry, Fırat University, Elazığ 23119, Türkiye; ealtintas@firat.edu.tr (E.A.); mcakgul@firat.edu.tr (M.C.A.)

**Keywords:** overlay restoration, preparation design, fracture resistance, marginal fit, 3D printing, additive manufacturing, zirconia-reinforced lithium silicate, CAD/CAM

## Abstract

Overlay restorations offer a conservative solution for teeth with substantial loss of tooth structure, but their success depends largely on the preparation design, material type, and fabrication technique. This study aimed to assess the effects of two different preparation designs and fabrication techniques on the fracture resistance and marginal adaptation of overlays fabricated from zirconia-reinforced lithium silicate (ZLS) and 3D-printed resin. Forty extracted human molars were randomly divided into two preparation design groups: occlusal reduction (O) and occlusal reduction with a round shoulder (OS). Each group was subdivided based on the material type: ZLS or 3D-printed resin (*n* = 10 per subgroup). Restorations were designed using CAD and manufactured using milling (ZLS) or additive manufacturing (3D-Printed). After cementation and thermomechanical aging (5500 cycles, 5–50 °C), marginal gaps were measured at 20 predefined points using scanning electron microscopy (SEM). The fracture resistance was tested using a universal testing machine. Data were analyzed using two-way ANOVA and post hoc tests (α = 0.05). The preparation design had a significant effect on both fracture resistance and marginal adaptation (*p* < 0.05). Group O showed significantly smaller marginal gaps than Group OS for both materials. The ZLS overlays exhibited a significantly higher fracture resistance than the 3D-printed resin overlays. All groups demonstrated marginal gaps within the clinically acceptable range (<120 μm). The fracture resistance and marginal adaptation of overlay restorations are significantly influenced by the preparation design and material type. A simpler occlusal reduction design results in better marginal adaptation, whereas round shoulder preparations provide a higher fracture resistance. Although the 3D-printed resin showed lower fracture resistance, its marginal adaptation was comparable to that of milled restorations, suggesting its potential as a conservative and cost-effective polymer composite alternative for digitally fabricated overlay restorations.

## 1. Introduction

The preservation of dental hard tissues constitutes a fundamental principle in restorative dentistry. In cases involving extensive loss of tooth structure, maintaining the maximum amount of sound tissue while ensuring the biological integrity and long-term viability of the restored tooth is particularly important [1]. However, the substantial loss of hard tissue that often follows restorative therapy compromises the structural strength of the tooth and significantly increases the risk of fracture [2]. Reduction in the remaining tooth structure decreases the resistance to occlusal loading and may negatively influence the long-term success of restorative procedures [3].

To reinforce teeth with compromised structural integrity, full-coverage crowns have traditionally been used [4]. Although increasing the thickness of the restoration can improve fracture resistance, conservation of a sound tooth structure remains a fundamental principle of prosthodontic treatment [5]. In cases where extensive tissue loss is present, restoring the tooth while maximally preserving the remaining structure is critically important for the success of restorative strategies [6].

With the growing emphasis on esthetics and minimally invasive dentistry, interest in conservative restorative approaches has increased substantially in recent years [7]. Partial coverage restorations address both esthetic and functional demands by targeting only defective or weakened areas, providing a more conservative alternative to full-crown preparations [8]. These restorations are commonly classified as inlays, onlays, or overlays based on the extent of surface coverage, and this classification serves as an important guide for treatment planning [9]. In posterior teeth, partial restorations contribute to long-term longevity by preserving the natural tooth structure while fulfilling esthetic and biomechanical requirements [8].

The success of restorative treatment largely depends on mechanical and biological factors such as fracture resistance and marginal adaptation [10]. The fracture resistance of overlay restorations is influenced by several parameters, including the physical properties of the restorative material, restoration thickness, luting protocol, and aging of both the tooth and restoration [11]. Among these factors, tooth preparation design is considered an important factor influencing the mechanical performance of indirect restorations, as it is associated with stress distribution within the tooth–restoration complex and the uniformity of the cement layer [12]. The preparation geometry has been reported to be related to stress distribution patterns and restoration seating accuracy, whereas different preparation designs have been associated with variations in marginal adaptation [11,13,14]. Furthermore, in digitally fabricated restorations, the preparation geometry has been shown to be associated with scanning accuracy and manufacturing precision, which are related to marginal adaptation and fracture resistance [15,16].

Recent studies have increasingly recommended ceramic occlusal veneers as a standard treatment option for severely distressed teeth [8,17]. CAD/CAM-fabricated ceramic occlusal veneers simplify the many technical steps associated with conventional fabrication methods [5]. Advances in intraoral scanning and milling technologies have enabled the production of more precise and detailed restorations [18]. Zirconia-reinforced lithium silicate (ZLS) is considered one of the most promising materials among modern CAD/CAM ceramics because of its favorable medium- and long-term durability, high mechanical strength, and excellent optical properties. It can be used as a monolithic restoration in posterior teeth or as an esthetic material in the anterior region, making it a widely accepted option for indirect restorations. Furthermore, ZLS has demonstrated adequate biomechanical behavior under occlusal forces even at reduced occlusal thicknesses of approximately 0.7 mm [19].

Recently, resin-based restorations fabricated using 3D printing technologies have become increasingly popular, particularly for provisional restorations and prototyping applications in digital dentistry [20]. Recently developed economical, high-strength, light-polymerized photopolymer resins have expanded the use of 3D-printed materials in permanent restorations [21]. Additive manufacturing offers advantages such as cost-effectiveness, production speed, and customization and positioning of 3D printing as a compelling option for prosthetic applications [22]. However, the long-term clinical performance of these materials, particularly in terms of fracture resistance and marginal adaptation, has not been sufficiently investigated [23].

Marginal adaptation is another critical parameter that affects the clinical longevity of restorations [24]. An inadequate marginal fit can lead to microleakage, bacterial infiltration, plaque accumulation, and secondary caries formation [25]. Although marginal gaps in the range of 50–120 µm are generally considered clinically acceptable, these values may vary dynamically depending on factors such as the thermal expansion characteristics of the material, luting protocol, design of tooth preparation and biomechanical stresses in the oral environment [26]. Literature evaluating the influence of preparation design and material type on marginal adaptation remains limited. Moreover, there is a need for further scientific evidence on how different preparation geometries and fabrication techniques (milled vs. 3D-printed) affect the marginal fit and fracture resistance of overlay restorations [27,28].

The novelty of the present study lies in the combined evaluation of marginal adaptation and fracture resistance of overlay restorations fabricated using subtractive CAD/CAM milling and additive manufacturing (3D printing) techniques with different preparation designs after thermomechanical aging. In addition, assessing the performance of additively manufactured polymer composite overlays in relation to preparation design provides both clinical insight and information on the structure–property relationships in polymer composite materials. Therefore, this study aimed to evaluate the effects of two different preparation designs on the fracture resistance and marginal adaptation of ZLS, and 3D-printed resin overlay restorations to support clinical decision-making in restorative design and fabrication protocols.

The null hypothesis of this study was that the preparation design has no significant effect on the marginal adaptation or fracture resistance of overlay restorations fabricated using milling or 3D printing technologies.

## 2. Materials and Methods

### 2.1. Study Design and Specimen Standardization

An overview of the materials used in this study is presented in Table 1. This in vitro study was performed at the Faculty of Dentistry, Bingöl University, following approval by the local Institutional Ethics Committee (Approval No: 2025-12/3).

Sample size was determined using the G*Power software (version 3.1.9.6; Heinrich-Heine-Universität Düsseldorf, Düsseldorf, Germany). Fracture resistance (N) was defined as the primary outcome, and marginal gap (µm) as the secondary outcome. The analysis was designed for a two-factor ANOVA model (two materials × two preparation designs) with a significance level set at α = 0.05 and a statistical power of 95%. An effect size of f = 0.40 (medium–large) was selected based on preliminary data obtained from a pilot study conducted in our laboratory, which demonstrated moderate to large differences in fracture resistance values between the preparation designs and material types. This effect size is consistent with the values reported in previous in vitro studies evaluating the mechanical outcomes of CAD/CAM restorations. Based on these parameters, a total sample size of 40 specimens (*n* = 10 per subgroup) was calculated and included in the study.

After obtaining written and verbal informed consent, 40 extracted human mandibular first molars with fully developed roots were included in this study. All teeth were extracted for periodontal indications, showed no visible caries or structural damage, and were stored in a 0.1% thymol–0.9% NaCl solution at room temperature for a maximum period of 30 days prior to use.

To ensure specimen standardization, the buccolingual and mesiodistal dimensions of each tooth were measured using a digital caliper, and only specimens with dimensional variations within 0.5 mm were accepted. The teeth were further inspected under ×10 magnification using an optical stereomicroscope (Leica S9 D, Leica Microsystems, Wetzlar, Germany) to confirm the absence of cracks, caries, or other defects. Any remaining periodontal ligament tissue and calculus deposits were carefully removed using manual instruments.

**Figure 1:**
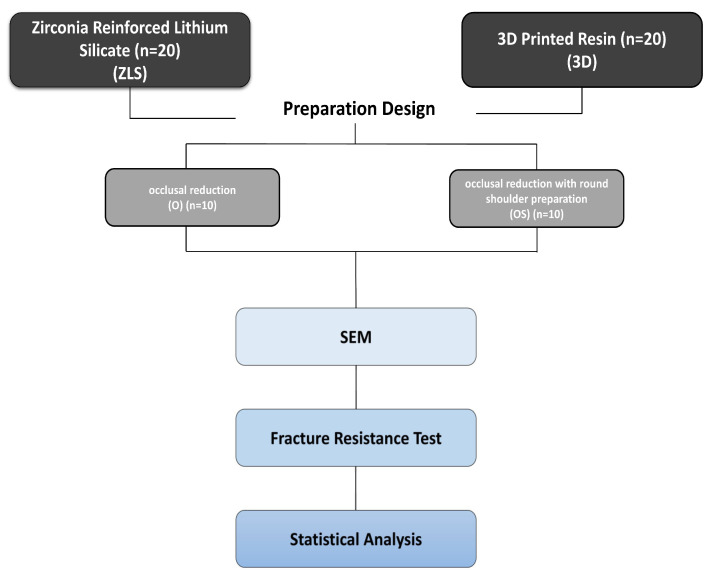
Study workflow illustrates the experimental design for the evaluation of CAD/CAM overlay materials.

Accordingly, 40 specimens were included to ensure adequate statistical power for the assessment of mechanical outcomes including marginal gap measurements and fracture resistance (Figure 1). For the fabrication of overlay restorations, the teeth were randomly assigned to two preparation design groups (*n* = 20) and further subdivided according to material type, as summarized in Table 2.

### 2.2. Tooth Preparation

**Figure 2:**
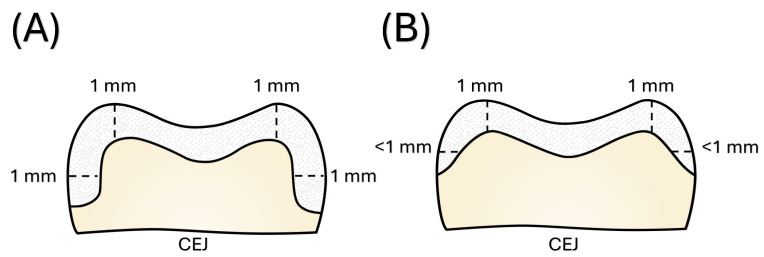
Schematic illustration of the occlusal reduction with round shoulder (**A**) and occlusal reduction (**B**) preparation designs applied in this study, with preparation margins located above the cementoenamel junction (CEJ).

In all specimens, occlusal reduction was performed to a depth of 1 mm following the natural anatomical morphology of the tooth (Figure 2). A silicone index was used throughout the procedure to verify the uniform reduction and maintain consistency among the samples. Preparations were performed using high-speed diamond burs (medium-grit, rounded-end tapered diamond bur; Komet Dental GmbH, Lemgo, Germany) under constant irrigation with a digital contra-angle handpiece (T2 Line, Dentsply Sirona, Bensheim, Germany), a new bur was used for each tooth. A cylindrical diamond bur was used to achieve a 1-mm occlusal reduction in all groups, followed by refinement with a fine-grit bur.

In the round shoulder group, a 90° shoulder finish line with a standardized depth of 1 mm and an axial divergence of approximately 6° was prepared at the supragingival level using a calibrated shoulder diamond bur (medium-grit, shoulder-end tapered diamond bur; Komet Dental GmbH, Lemgo, Germany). The shoulder depth was verified using the silicone index and periodically checked using a periodontal probe to ensure dimensional accuracy. Axial divergence was controlled by maintaining consistent a handpiece angulation according to visual guidance throughout the preparation process. All preparations were performed by the same operator to minimize inter-sample variability and ensure the uniformity of the preparation geometry. To enhance restoration–substrate compatibility and maintain a uniform cement layer, all prepared surfaces were smoothed using polishing discs (Sof-Lex™ Pop-On; 3M ESPE, St. Paul, MN, USA).

### 2.3. Restoration Design and Fabrication

**Figure 3:**
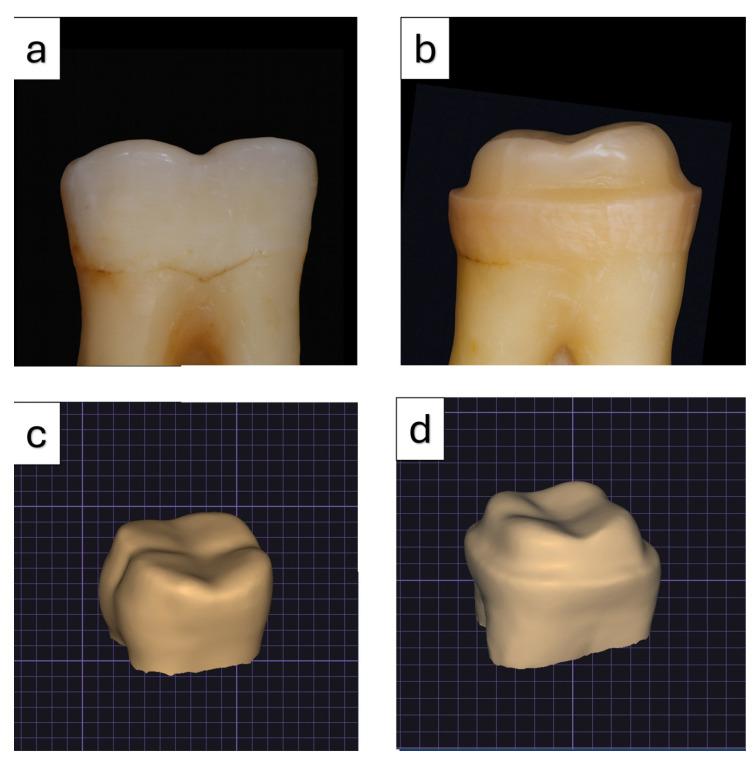
Panels (**a**,**b**) illustrate the teeth prepared with anatomical occlusal reduction and occlusal reduction with round shoulder, respectively, while panels (**c**,**d**) represent the corresponding STL files obtained from digital scanning of these preparations.

After tooth preparation, all specimens were digitized using an intraoral scanning system (Trios 4; 3Shape, Copenhagen, Denmark), and the resulting datasets were saved in the stereolithography (STL) format for subsequent digital processing (Figure 3). Restorations were designed using CAD software (Exocad v3.0; Exocad GmbH, Darmstadt, Germany) with anatomically standardized contours. A consistent restoration thickness of 1 mm was defined for all designs, and the cement space parameter was uniformly set at 50 µm. Identical design settings were applied to each specimen to ensure consistency during the fabrication process.

Zirconia-reinforced lithium silicate (ZLS) restorations were manufactured using a five-axis milling unit (Ceramill Motion 2; Amann Girrbach, Koblach, Austria). Following fabrication, all restorations were visually examined for potential defects, and specimens that did not meet the quality criteria were remanufactured. The same STL design files were subsequently used for the production of polymer-based restorations using additive manufacturing. Prior to the printing process, the build platform and printing materials were prepared in strict accordance with the manufacturer’s recommendations to ensure standardized fabrication conditions.

Resin overlay restorations were produced using a stereolithography (SLA)-based Formlabs 3D printer (Formlabs 3; Formlabs Inc., Somerville, MA, USA) with the appropriate photopolymer resin. All resin restorations were printed with a standardized build orientation, with the occlusal surface positioned parallel to the build platform. The same printing orientation was applied to all specimens to minimize the variability related to the interlayer bonding and anisotropic mechanical behavior.

After printing, the specimens were washed in 95% isopropyl alcohol for 3 min using the Form Wash unit, then post-cured in the Form Cure UV unit (Formlabs Inc., Somerville, MA, USA), which utilizes multidirectional LED light at a wavelength of 405 nm, at 60 °C for 20 min, as recommended by the resin manufacturer to achieve optimal mechanical properties. After removing the support structures, an additional curing cycle was performed under the same parameters to ensure the dimensional stability of the fitting surfaces. Restorations that did not meet quality criteria were reprinted.

### 2.4. Cementation Procedure

Prior to the cementation procedure, all restorations were subjected to ultrasonic cleaning in 99% isopropanol for 3 min using an ultrasonic device (Ultrasonic Cleaner UC-20; Biobase, Jinan, China). The prepared tooth surfaces were cleaned with fluoride-free pumice, thoroughly rinsed with water, and gently air-dried.

For zirconia-reinforced lithium silicate (ZLS) restorations, the internal surfaces were conditioned with 5% hydrofluoric acid (IPS Ceramic Etching Gel; Ivoclar Vivadent AG, Schaan, Liechtenstein) for 20 s, followed by rinsing with water for 60 s and air drying. A silane coupling agent (Monobond S; Ivoclar Vivadent AG, Schaan, Liechtenstein) was then applied, after which a universal adhesive (All-Bond Universal; Bisco Inc., Schaumburg, IL, USA) was applied and air-dried for 60 s.

The intaglio surfaces of 3D-printed resin restorations were conditioned according to the manufacturer’s instructions. The surface treatment consisted of airborne-particle abrasion with 50 µm aluminum oxide particles (Cobra; Renfert, Hilzingen, Germany) at a pressure of 0.15 MPa, followed by the application of a universal adhesive (All-Bond Universal), which was air-dried without light activation.

The tooth substrates were etched with 37% phosphoric acid (N-Etch; Ivoclar Vivadent AG, Schaan, Liechtenstein) for 30 s on enamel and 15 s on dentin, rinsed thoroughly, and gently dried. Subsequently, a universal adhesive (All-Bond Universal) was applied to the tooth surfaces according to the manufacturer’s recommendations.

All restorations were luted using a dual-cure resin cement (Variolink Esthetic DC; Ivoclar Vivadent AG, Schaan, Liechtenstein). To facilitate clear distinction between the tooth structure and cement layer during marginal evaluation, a contrasting shade of the resin cement was selected. The restorations were seated using manual finger pressure applied in a standardized manner by a single experienced operator to simulate clinical cementation conditions and reflect routine clinical cementation procedures. To minimize operator-related variability, the same seating technique and pressure were consistently applied to all specimens until complete seating was visually confirmed, and excess cement was carefully removed prior to polymerization. Each surface was light-cured for 20 s using a light-curing unit with an output intensity of 650 mW/cm^2^ (DTE O-Light Woodpecker; Guilin, China). After cementation, the margins were polished using a flexible polishing disc. The specimens were stored in water at room temperature until further testing was performed.

To simulate periodontal ligament behavior and enhance the physiological relevance of the experimental model, the root surfaces were coated with a 0.2–0.3 mm layer of vinyl polysiloxane elastomer (Elite HD+; Zhermack, Badia Polesine, Italy). The specimens were subsequently embedded in polyvinyl chloride cylinders using self-curing acrylic resin, with the embedding level positioned 2 mm apical to the cementoenamel junction. This setup was intended to promote a more realistic stress distribution during thermomechanical aging and fracture resistance testing.

### 2.5. Thermomechanical Aging

To approximate the intraoral functional conditions, all specimens were subjected to a combined thermomechanical aging protocol using a chewing simulator equipped with an integrated thermocycling unit (CS-4.8 Professional Line; SD Mechatronik GmbH, Feldkirchen-Westerham, Germany). Mechanical and thermal stresses were applied simultaneously to reproduce the complex loading conditions encountered in the oral environment.

**Figure 4:**
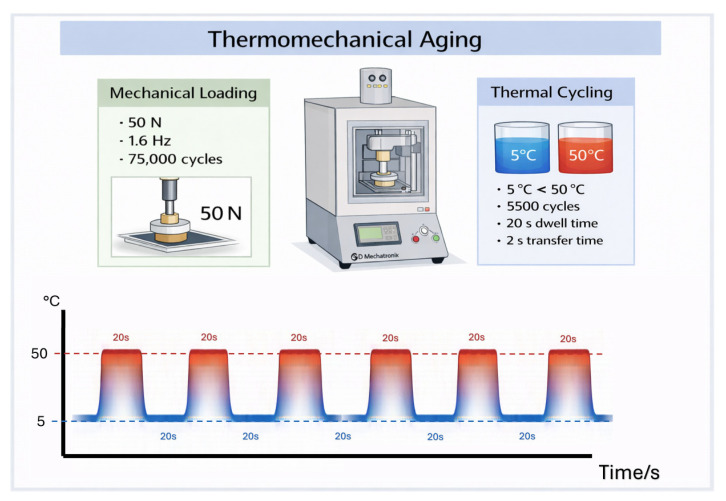
Schematic representation of the thermomechanical aging procedure used in this study.

Mechanical fatigue was induced by applying a vertical load of 50 N at a frequency of 1.6 Hz for 75,000 cycles, corresponding to approximately 6–12 months of clinical mastication. At the same time, thermal cycling was carried out by alternating temperatures between 5 °C and 50 °C within the same chamber. Each temperature bath was maintained for 20 s, with a transfer time of 2 s, resulting in a total of 5500 thermal cycles (Figure 4). This protocol ensured the continuous exposure of the specimens to combined mechanical loading and thermal fluctuations.

Following the aging process, all specimens were stored in an isotonic saline solution at room temperature for 24 h before marginal gap assessment and fracture resistance testing. Subsequently, the specimens were visually inspected for macroscopic cracks under standardized lighting conditions using ×10 magnification with an optical stereomicroscope.

### 2.6. Marginal Gap Measurement

Marginal adaptation was determined by evaluating the vertical marginal discrepancy between the restoration margin and prepared tooth surface using scanning electron microscopy (SEM). Before analysis, all specimens were sputter-coated with a thin gold–palladium layer using a sputter-coating device (Emitech K550 Sputter Coater; Quorum Technologies Ltd., Lewes, UK) to enhance surface conductivity and improve image quality.

**Figure 5:**
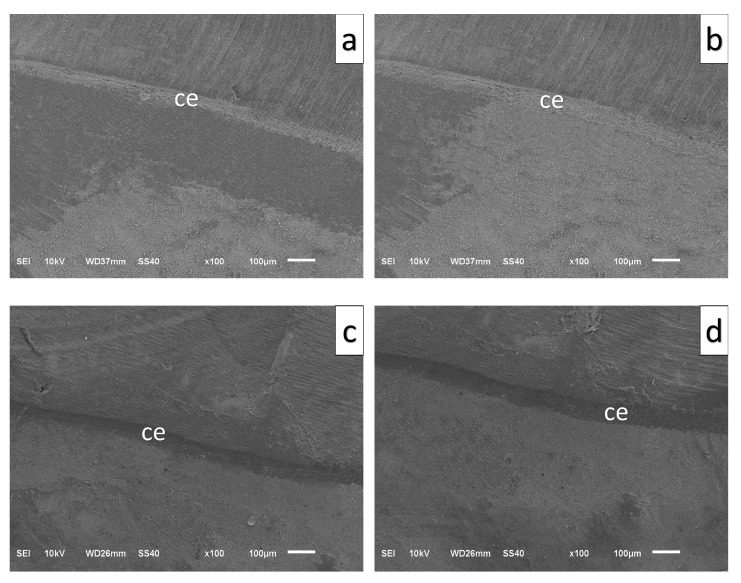
High magnification scanning electron microscopy images of the border between crown, cement and tooth structure. (**a**) O—3D-Printed, (**b**) OS—3D-Printed, (**c**) O—ZLS, (**d**) OS—ZLS, ce: Cement.

For each specimen, the marginal gap values were recorded at 20 standardized reference locations distributed equally along the buccal, lingual, mesial, and distal aspects of the restoration margin. Scanning electron microscopy observations were performed using a JEOL JSM-6510 microscope (JEOL Ltd., Tokyo, Japan) operated at an accelerating voltage of 10 kV. All measurements were obtained at a fixed magnification of 100× by a single experienced examiner to minimize measurement-related variability. The average of the 20 recorded values was calculated and the marginal gap for each specimen was considered (Figure 5).

To verify measurement consistency, a subset of specimens was randomly selected and re-evaluated. Intra observer reliability was assessed using the intraclass correlation coefficient (ICC), which yielded values of ≥0.90.

### 2.7. Fracture Resistance Test

**Figure 6:**
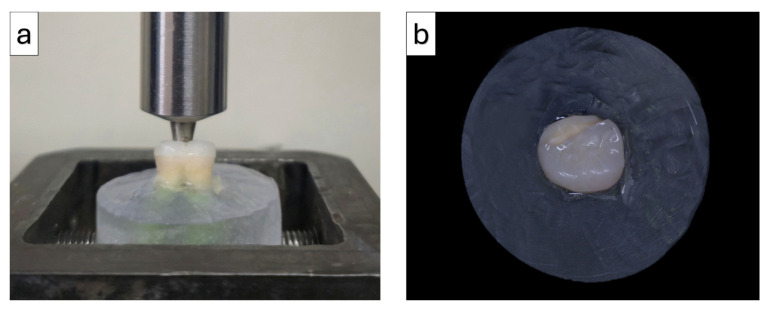
Experimental setup for fracture resistance testing of overlay restorations: (**a**) loading configuration in the universal testing machine; (**b**) occlusal view of the embedded specimen after testing.

The fracture resistance of all the specimens was evaluated using a universal testing machine fitted with a 10 kN load cell (Model AGS-X; Shimadzu Corporation, Kyoto, Japan). Testing procedures were carried out under controlled room temperature conditions (23 ± 2 °C). Each specimen was positioned securely in the testing apparatus, and a compressive force was applied vertically at a constant crosshead speed of 0.5 mm/min. The load was delivered to the occlusal surface at the central fossa using a stainless-steel indenter with a diameter of 5 mm. The maximum load recorded at the point of fracture was measured in Newtons (N) and expressed as mean ± standard deviation for each experimental group (Figure 6).

### 2.8. Statistical Analysis

Data analysis was performed using statistical software (IBM SPSS Statistics, version 20.0; IBM Corp., Armonk, NY, USA). Descriptive statistics were calculated for all measured variables and are presented as mean values with corresponding standard deviations. The outcome measures included marginal gap and fracture resistance.

The distribution of the data was examined using the Shapiro–Wilk test, and variance homogeneity was evaluated with Levene’s test. As both assumptions were satisfied, parametric statistical methods were applied. Given the factorial structure of the experimental design, two-way analysis of variance (ANOVA) was used to investigate the effects of preparation design and material type, as well as their potential interaction, on marginal gap and fracture resistance outcomes.

Where statistically significant differences were identified, Tukey’s honestly significant difference (HSD) test was employed for multiple comparisons. Independent sample t-tests were conducted in situations requiring direct comparison between the two groups. Statistical significance was set at *p* < 0.05 for all analyses.

## 3. Results

Following thermomechanical aging, no macroscopic cracks or fractures were observed in any of the specimens, indicating that both restorative materials preserved their structural integrity under the combined thermal and mechanical loading conditions.

### 3.1. Marginal Gap Analysis

#### 3.1.1. Fabrication Technique

Statistical analysis demonstrated that the fabrication technique had no significant effect on the marginal gap values (milling vs. 3D printing; two-way ANOVA, *p* > 0.05). Furthermore, when comparisons were performed within the same preparation design, no significant differences in the marginal gap values were detected between the material types (ZLS vs. 3D-printed resin; *p* > 0.05). For the occlusal-only preparation group (O), the mean marginal gap values were 59.07 ± 11.52 µm for 3D-printed and 63.21 ± 14.60 µm for ZLS restorations, whereas in the occlusal plus shoulder preparation group (OS), the corresponding values increased to 80.61 ± 16.75 µm for 3D-printed and 84.52 ± 18.25 µm for ZLS restorations (Table 3). All the experimental groups showed marginal gap values within the clinically acceptable range (<120 µm).

Although the differences were not statistically significant, restorations fabricated using additive manufacturing tended to exhibit lower marginal gap values. The highest mean marginal gap values were recorded in the ZLS groups.

#### 3.1.2. Preparation Design

The preparation design significantly affected the marginal gap values, regardless of the fabrication technique or material type (two-way ANOVA, *p* < 0.05). Specimens prepared with occlusal reduction (O) demonstrated significantly lower marginal gap values compared with those prepared with an occlusal reduction combined with a round shoulder (OS) design in both material groups (*p* < 0.05). The corresponding mean ± standard deviation values are summarized in Table 4.

Pairwise effect size calculations showed variability in the magnitude of group differences, with Cohen’s d values ranging from small to very large (0.23–1.37; Table 5).

**Figure 7:**
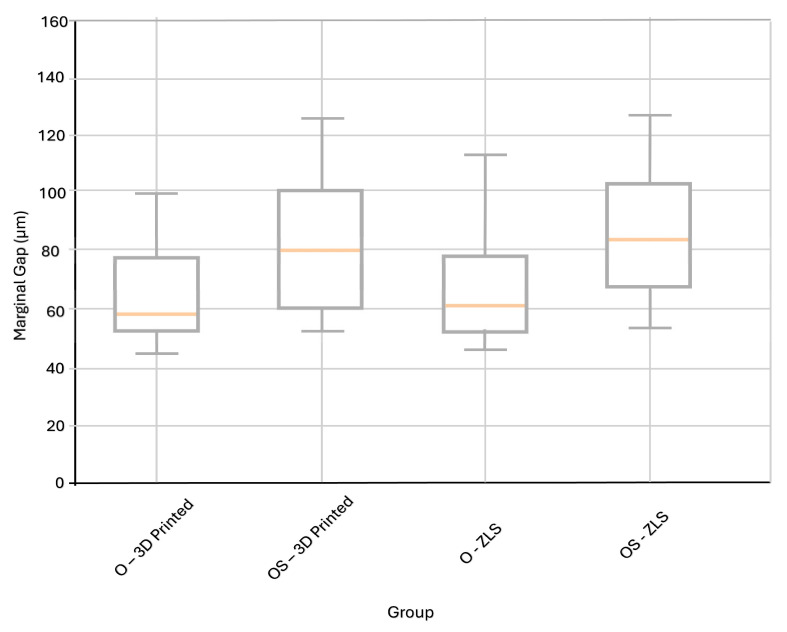
Distribution of marginal discrepancy (µm) values for ZLS and 3D-printed restorations in occlusal reduction (O) and occlusal reduction with round shoulder preparation (OS) conditions, illustrated using box plots.

Due to the high number of marginal gap measurements collected for each group, box plot representation was selected to illustrate the distributional characteristics of the data (Figure 7). This graphical method provides insight into central tendency and variability, thereby supporting the evaluation of marginal adaptation across different groups.

### 3.2. Fracture Resistance

#### 3.2.1. Fabrication Technique

**Figure 8:**
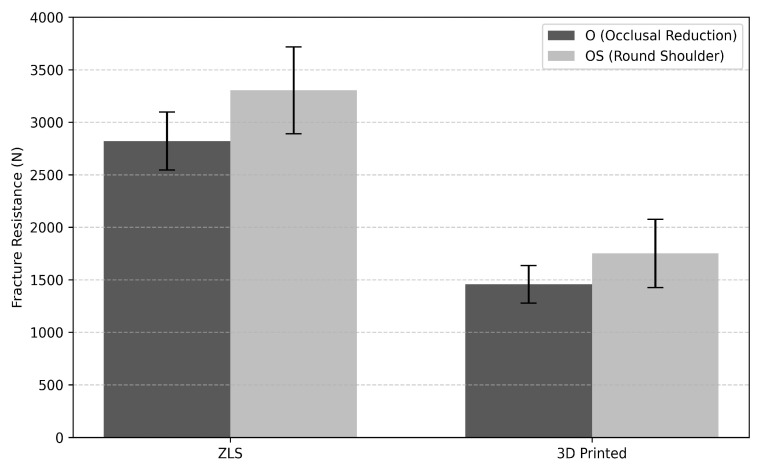
Comparison of mean fracture resistance values (N) of ZLS and 3D-printed overlay restorations according to preparation design. Error bars represent standard deviations.

When data from both preparation designs were analyzed collectively, the fabrication technique and material type were found to have a significant effect on fracture resistance outcomes, independent of the preparation design (*p* < 0.05). Across both preparation designs, milled restorations demonstrated significantly higher fracture resistance values than did 3D-printed restorations. The lowest mean fracture resistance values were recorded in the 3D-printed occlusal reduction group. For the occlusal preparation group (O), the mean fracture resistance was 2821.30 ± 275.51 N for ZLS restorations and 1456.97 ± 178.65 N for 3D-printed restorations, whereas in the occlusal plus shoulder preparation group (OS), the corresponding mean values were 3303.76 ± 412.52 N for ZLS and 1751.50 ± 325.83 N for 3D-printed restorations (Table 6, Figure 8).

#### 3.2.2. Preparation Design

The preparation design had a statistically significant influence on fracture resistance outcomes, independent of the fabrication technique or material type (*p* < 0.05). The mean fracture resistance was 2125.05 ± 143.54 N for restorations prepared with occlusal reduction only (O), whereas restorations with round shoulder preparation (OS) exhibited a higher mean fracture resistance of 2525.37 ± 160.73 N (Table 7). Across both material types, restorations with a round shoulder preparation exhibited higher mean fracture resistance values than those prepared with occlusal reduction alone (*p* < 0.05).

In addition to statistical significance, the effect size analysis demonstrated large to very large differences among the experimental groups, as indicated by Cohen’s d values ranging from 1.12 to 5.87 (Table 8).

## 4. Discussion

In this study, the influence of different preparation designs on the marginal gap values and fracture resistance of milled and 3D-printed overlay crowns was evaluated. The findings demonstrated that preparation geometry had a significant influence on both parameters, irrespective of the restorative material. The absence of significant interaction between material type and preparation design indicated that the observed effects on marginal adaptation and fracture resistance were primarily driven by the main factors rather than their combined influence. Therefore, the null hypothesis stating that the preparation design would not affect marginal adaptation and fracture resistance was rejected. These results are consistent with previous reports emphasizing the critical role of preparation configuration in the marginal fit and mechanical behavior of indirect restorations [5,13,14].

Preparations involving only occlusal reduction exhibited lower marginal gap values than those involving a round shoulder. This may be attributed to the simplified geometry, which facilitates digital scanning and enhances precision throughout the CAD/CAM fabrication workflow [15].

To simulate clinical conditions as closely as possible in an in vitro setting, extracted human teeth were used, and all preparation, impression, fabrication, and cementation procedures were performed in accordance with standard clinical protocols. Cementation was performed using manual finger pressure, and all procedures were performed by a single clinician to minimize operator-related variability. Temperature changes in the oral environment may influence the performance of restorations [29]. Yılmaz et al. [30] reported significant differences in marginal gaps between milled and additively manufactured crowns after aging. Accordingly, thermal aging in the current study was designed to simulate approximately six months of clinical service.

The marginal gap is defined as the vertical discrepancy between the cervical margin of the restoration and the finish line, representing the exposed surface area of the luting cement [31]. Based on this definition, marginal adaptation in the present study was assessed using direct SEM imaging, which is consistent with the methodology reported in previous studies [30,32]. Direct SEM assessment is widely preferred in literature owing to its advantages including elimination of specimen sectioning, compatibility with subsequent fracture testing, time efficiency, and reduced measurement errors [33]. Other methods such as silicone replica, stereomicroscopy, and optical microscopy have also been described in the literature [34,35]. Although the silicone replica technique allows for the assessment of internal adaptation under SEM, the thin elastomeric layer is prone to deformation during removal and may be affected by thermal aging, thereby limiting its reliability [36,37].

The results of this study demonstrate that the marginal gap values varied significantly according to the preparation design. The smallest gaps were observed in the specimens prepared with only occlusal reduction. This may be attributed to the simplified geometry, which facilitates digital scanning and improves the accuracy of the manufacturing process. In contrast, the deeper axial reduction required for round shoulder preparations may increase cement space and water sorption, thereby negatively affecting marginal adaptation [38]. Supporting these findings, Sirous et al. [11] reported that preparations with fewer internal line angles and simpler contours improve the efficiency of the digital workflow and reduce marginal discrepancies. Similarly, Fuzzi et al. [16] observed that flatter preparations with fewer internal angles promoted more uniform cement flow during cementation, resulting in an improved marginal fit. In this context, the large effect sizes observed between the preparation designs in the present study suggest that simplified occlusal reduction may provide a more predictable marginal adaptation in clinical practice.

All specimens in the present study exhibited marginal gap values greater than the predetermined cement spacer of 50 µm. This deviation may be attributed to the intrinsic 50-µm layer thickness associated with 3D printing or the 1.8-mm diameter of the milling burs used in the subtractive fabrication process. Nonetheless, all measured values remained within clinically acceptable limits (<120 µm), which is consistent with previous reports [39,40]. Although no statistically significant difference was observed, 3D-printed restorations tended to exhibit slightly lower marginal gap values, corroborating earlier findings [30,41,42].

However, the existing literature presents inconsistent findings regarding the influence of fabrication technique on marginal adaptation. Igret et al. [41] reported that milled provisional crowns exhibited superior marginal adaptation compared to 3D-printed crowns; however, they emphasized that the reduced material waste and cost-effectiveness of additive manufacturing make them a promising option for future clinical use. Falahchai et al. [13] further demonstrated that the preparation design significantly influenced the marginal gap values in overlay restorations, with the lowest gaps observed in preparations involving only occlusal reduction. As the preparation complexity increased, the marginal discrepancies also increased. Additionally, the hydraulic pressure generated by the luting agent may impede the complete seating of restorations in complex preparations, contributing to marginal misfits [43,44]. Differences across studies may result from variations in milling bur dimensions, printer resolution, or manufacturing parameters.

Minimally invasive preparation designs that preserve a greater amount of sound tooth structure have gained substantial clinical interest [45]. Several in vitro studies have demonstrated that high-strength ceramic occlusal veneers provide predictable and favorable outcomes for the restoration of worn occlusal surfaces [46]. Furthermore, conservative occlusal preparations have consistently been shown to improve marginal adaptation and enhance the biomechanical performance of restorations, while maintaining the tooth structure [11,47].

Albelasy et al. [48] reported that both the material type and restoration thickness significantly affect the fracture resistance of CAD/CAM restorations, and previous studies have identified occlusal ceramic fractures as a predominant cause of restoration failure. Despite their clinical relevance, studies that directly compare different preparation designs for digitally fabricated overlay restorations are limited [5].

In this study, the fracture resistance values obtained for the tested occlusal overlays were comparable with those reported in the literature. Al-Akhali et al. [46] reported a mean fracture resistance of approximately 1667 N for zirconia-reinforced lithium silicate overlays with an occlusal thickness of 0.5–0.8 mm, emphasizing that both ceramic thick-ness and preparation design play critical roles in fracture behavior. Likewise, Yıldız et al. [49] reported clinically acceptable fracture resistance values for 1-mm lithium disilicate laminate veneers.

Zirconia-reinforced lithium disilicate was selected for this study because of its superior mechanical properties and favorable esthetic performance. SLA-based 3D printing systems have gained increasing use in dentistry owing to their high resolution and relatively isotropic mechanical behavior [50]. Although the early generations of printed resin materials are primarily limited to provisional restorations owing to their inadequate strength, recent advancements in photopolymer resin technology have produced materials with promising potential for permanent restorations [51,52].

Suksuphan et al. [53] compared milled and 3D-printed resin crowns with a 1-mm occlusal thickness and reported that the material type significantly influenced both marginal fit and fracture resistance. Their findings indicated that printed crowns demonstrated superior marginal adaptation, whereas all tested crowns provided adequate fracture resistance, even at minimal thickness. Similarly, Al-Halabi et al. [54] concluded that both milled, and 3D-printed primary crowns offer clinically acceptable esthetic and functional outcomes for restoring endodontically treated primary molars with satisfactory marginal adaptation and retention.

After thermal aging, the mean fracture resistance values in the O group were 1456 and 2821 N for the 3D-printed resin and ZLS crowns, respectively. In the OS group, the corresponding values were 1751 N and 3303 N, respectively. Given that physiological occlusal forces typically reach approximately 100 N during mastication and may increase to 320 N during maximal intercuspation [55], the values recorded in this study greatly exceed functional requirements. These findings indicated that the tested restorations provided sufficient resistance to occlusal loading under clinical conditions.

Zirconia-reinforced lithium disilicate demonstrated a significantly higher fracture resistance than the 3D-printed resin across all preparation designs. This difference can primarily be attributed to the superior mechanical properties of the ZLS, including its high flexural strength (>400 MPa) and hardness [56]. In contrast, polymer-based 3D-printed materials generally exhibit lower fracture resistance, which may be discussed within the framework of polymer composite behavior [57,58]. Unlike ceramic materials, polymer-based composites exhibit viscoelastic characteristics and a lower elastic modulus, which may contribute to differences in the stress distribution within the tooth–restoration complex. In addition, layer-by-layer fabrication inherent to additive manufacturing may influence the polymer chain orientation and crosslink density, factors that have been reported to affect mechanical performance, including fracture resistance and marginal stability [59]. Nevertheless, although the fracture resistance of the tested 3D-printed resin was lower than that of the ZLS, all values exceeded the physiological occlusal force levels.

The mechanical behavior and fracture resistance of additively manufactured resin restorations are influenced by printing orientation due to anisotropic properties and interlayer bonding characteristics. Numerous in vitro studies have reported significant differences in the fracture strength among specimens fabricated using different build orientations [60]. Alkhateeb et al. [61] reported the highest fracture resistance in specimens printed in occlusal orientation. In the present study, an occlusal-oriented printing configuration was deliberately selected to minimize the potential influence of printing orientation and interlayer anisotropy on the fracture resistance of 3D-printed resin restorations, thereby standardizing the specimen geometry and reducing orientation-related variability. Therefore, within the limitations of this in vitro study, additively manufactured polymer-based overlays may be considered for further investigation as potential restorative materials.

Numerous studies have shown that preparation design has a direct impact on the fracture resistance of partial-coverage restorations. Minimally invasive preparations and preservation of marginal ridges are associated with a lower fracture incidence [6,11,62]. Excessive removal of the tooth structure, particularly in central regions such as the mesio-occlusal–distal areas, may compromise the mechanical integrity of the tooth [6]. Therefore, the preservation of the natural tooth structure plays a critical role in enhancing fracture resistance and supporting long-term restoration success. More complex geometries may create localized stress concentrations, reducing the fracture resistance of both the restoration and tooth–restoration complex [63].

It should be acknowledged that specimen preparation procedures prior to SEM analysis, including sectioning and dehydration under vacuum conditions, may influence the measured marginal gap values. Cutting procedures may introduce minor artifacts at the restoration margins, whereas drying processes could potentially affect the dimensional stability of the luting cement or tooth structure. However, in the present study, all specimens were subjected to identical preparation protocols, and the measurements were performed under standardized conditions. Therefore, although absolute marginal gap values may have been influenced to some extent by specimen preparation, the comparative analysis among groups remains reliable.

In addition to measurement-related considerations, certain methodological aspects of study design should also be considered when interpreting the present findings. It should be acknowledged that in the present study, fabrication the technique was inherently linked to material type, as zirconia-reinforced lithium silicate restorations were produced by milling, whereas polymer-based restorations were fabricated using additive manufacturing. This coupling may be considered a potential confounding factor when interpreting the isolated effects of materials and fabrication techniques. Future studies evaluating identical materials fabricated using different manufacturing techniques would further clarify the independent influence of the fabrication methods.

Several limitations of the present study may have influenced the clinical interpretation of our findings. These include the lack of evaluation of internal adaptation, a relatively small sample size, and the inability to fully reproduce intraoral conditions. In addition, fracture behavior was assessed solely by recording the maximum load at failure during fracture resistance testing, and no detailed analysis or imaging of fracture failure patterns was performed. Moreover, variations in the age and morphological characteristics of the extracted human teeth used in this study may have introduced challenges in achieving complete standardization among the groups. The relatively small sample size and the inherent anatomical variability of extracted human teeth may have contributed to the variation observed in the results and should therefore be considered when interpreting the findings of the present study.

## 5. Conclusions

Within the limitations of this in vitro study, the results demonstrated that marginal gap values of overlay restorations were not significantly influenced by fabrication technique or material type, whereas preparation design had a significant effect, with occlusal reduction, with only preparations showing lower marginal gap values than round shoulder designs. Fracture resistance was significantly affected by both fabrication technique and preparation design, with milled restorations and round shoulder preparations exhibiting higher fracture resistance values. No macroscopic cracks or fractures were observed following thermomechanical aging, indicating that both restorative materials maintained their structural integrity under combined thermal and mechanical loading conditions. The effect size analysis further supported the magnitude of the observed differences among the experimental groups, particularly for the fracture resistance outcomes.

## Figures and Tables

**Table 1:** Materials used in this study.

Material	Product Name	Composition	Manufacturer
Zirconia-Reinforced Lithium Silicate Glass-ceramic (ZLS)	Vita Suprinity^®^ PC	SiO_2_: 56–64% Li_2_O: 15–21% ZrO_2_: 8–12% TiO_2_: ~10% Coloring pigments: <10%	VITA Zahnfabrik H. Rauter GmbH, Bad Säckingen, Germany
3D-Printed Resin	Crowntec^®^	BisEMA (Bisphenol A polyethylene glycol diether dimethacrylate): 50–<75% Methyl benzoylformate: 1–<5% TPO-type photoinitiator: 1–<5%	Saremco Dental AG, Rebstein, Switzerland

**Table 2:** Experimental groups according to preparation design and material type.

Group Code	Preparation Design	Material Type	*n*
O-ZLS	Occlusal reduction	Zirconia-reinforced lithium silicate	10
O-3D-Printed	Occlusal reduction	3D-printed resin	10
OS-ZLS	Occlusal reduction with round shoulder	Zirconia-reinforced lithium silicate	10
OS-3D-Printed	Occlusal reduction with round shoulder	3D-printed resin	10

**Table 3:** The descriptive and comparative statistics of the marginal gaps values (µm) of the restoration types.

Group	Restoration Type (*n* = 10)	Mean (±SD)	95% Confidence Interval for Mean		Minimum Value	Maximum Value
			Lower Bound	Upper Bound		
O	ZLS	63.21 ± 14.60	52.77	73.65	53.81	73.37
	3D-Printed	59.07 ± 11.52	50.83	67.31	49.97	70.41
OS	ZLS	84.52 ± 18.25	71.47	97.58	71.22	96.46
	3D-Printed	80.61 ± 16.75	68.63	92.59	68.46	92.53

SD: Standard deviation.

**Table 4:** The descriptive and comparative statics of the marginal gaps values (µm) of the preparation types.

Preparation Type (*n* = 20)	Mean (±SD)	95% Confidence Interval for Mean		Minimum Value	Maximum Value
		Lower Bound	Upper Bound		
O	61.50 ± 15.15	54.41	68.59	49.97	73.37
OS	82.25 ± 16.05	74.73	89.77	68.46	92.53

SD: Standard deviation.

**Table 5:** Cohen’s d values for pairwise group comparisons (marginal gap).

Group Comparison	Cohen’s d	Effect Size Interpretation
O-ZLS vs. O-3D-Printed	0.321	Small effect
OS-ZLS vs. OS-3D-Printed	0.230	Small effect
O-ZLS vs. OS-ZLS	1.373	Very large effect
O-3D-Printed vs. OS-3D-Printed	1.121	Large effect

d values of <0.5 small, 0.5–0.8 medium, ≥0.8 large, ≥1.2 very large.

**Table 6:** The descriptive and comparative statics of the Fracture Resistance (Newton) of the restoration types.

Group	Restoration Type (*n* = 10)	Mean (±SD)	95% Confidence Interval for Mean		Minimum Value	Maximum Value
			Lower Bound	Upper Bound		
O	ZLS	2821.30 ± 275.51	2683.63	2916.31	2461.07	3147.12
	3D-Printed	1456.97 ± 178.65	1362.09	1537.87	1234.59	1672.34
OS	ZLS	3303.76 ± 412.52	3176.52	3423.01	2942.28	3664.91
	3D-Printed	1751.50 ± 325.83	1642.84	1857.23	1473.56	2030.39

SD: Standard deviation.

**Table 7:** The descriptive and comparative statics of the Fracture Resistance (Newton) of the preparation types.

Preparation Type (*n* = 20)	Mean (±SD)	95% Confidence Interval for Mean		Minimum Value	Maximum Value
		Lower Bound	Upper Bound		
O	2125.05 ± 143.54	2049.28	2200.72	1234.59	3147.12
OS	2525.37 ± 160.73	2444.81	2605.19	1473.56	3664.91

SD: Standard deviation.

**Table 8:** Cohen’s d values for pairwise group comparisons (fracture resistance).

Group Comparison	Cohen’s d	Effect Size Interpretation
O-ZLS vs. O-3D-Printed	4.174	Very large effect
OS-ZLS vs. OS-3D-Printed	5.877	Very large effect
O-ZLS vs. OS-ZLS	1.373	Very large effect
O-3D-Printed vs. OS-3D-Printed	1.121	Large effect

d Values of <0.5 small, 0.5–0.8 medium, ≥0.8 large, ≥1.2 very large.

## Data Availability

The original contributions presented in this study are included in the article. Further inquiries can be directed to the corresponding author.
